# Supplementary material for: Low Iron Diet Improves Clinical Arthritis in the Mouse Model of Collagen-Induced Arthritis
Source: Cells. 2024 Oct 29;13(21):1792. doi: 10.3390/cells13211792 (PMC11545767; doi:10.3390/cells13211792)
Supplement: Supplementary file 1 [file cells-13-01792-s001.zip › cells-3058355-supplementary.pdf]

Supplementary Material

Low iron diet improves clinical arthritis in the mouse model of collagen-induced arthritis

Godehard A. Scholz[1], Sisi Xie[1], Tasneem Arsiwala[1], Daniel Guggisberg[1], Monique Vogel[1], Martin Bachmann[1], Burkhard Möller[1]

[1] Department for Rheumatology and Immunology, Inselspital, University Hospital Bern, Bern, Switzerland

Supplementary Table S1: Forward and reverse primers of the respective genes.

Gene	Forward primer 5′-3′	Reverse primer 5′-3′
FTH1	CCGAGATGATGTGGCTCTGA	AGTCATCACGGTCTGGTTTCTTTA
TFRC	GAGCTATAAGCTTTGGGTGGGA	TGCTACAAGGGAGTACTAGGAAG
FPN1	GGTGGTTCAGAATGTGTCCGTCATC	GGATTGTGATCGCAGTGGCAGTAC
VEGF	TCCGTAGTAGCCGTGGTCTGC	CCCTCTCCTCTTCCTTCTCTTCCTC
SDF1	CATCGCCAGAGCCAACGTCAAG	AGCCGTGCAACAATCTGAAGGG
GPX4	GCCTGGATAAGTACAGGGGTT	CATGCAGATCGACTAGCTGAG
ACSL4	CTGATCTGCCTCCTGACGTTTGGAA	ACAACGTCTTGGCGTCTGAGAAGT
SLC7A11	TTGGAGCCCTGTCCTATGC	CGAGCAGTTCCACCCAGAC
SLC3A2	GCAGGACGGTGTGGATGGTTTC	GCAGGTCGCTGGTGGATTCAAG
RPS29	GTCTGATCCGCAAATACGGG	AGCCTATGTCCTTCGCGTACT

FTH1: ferritin heavy chain 1, TFRC: transferrin receptor protein 1, FPN1: ferroportin-1, VEGF: vascular endothelial growth factor, SDF1: stromal cell-derived factor 1, GPX4: glutathione peroxidase 4, ACSL4: acyl-CoA synthetase long chain family member 4, SLC7A11: solute carrier family 7 member 11, SLC3A2: solute carrier family 3 member 2, RPS29: ribosomal protein S29

Supplementary Table S2: Weight development (g) during the experimental course shown as mean per cage in mice fed (A) a normal iron diet and (B) a low iron diet. The results are presented graphically below the tables. The term "round" refers to a single independent experiment. In round 6, two cages were operated in parallel for each condition, which is differentiated by "a" and "b".

A. Normal iron diet.

	Round 1	Round 2	Round 3	Round 4	Round 5	Round 6a	Round 6b
**Day 0**	17.26	20.06	21.04		26.74	21.84	22.58
**Day 10**				26.88			
**Day 16**				26.38			
**Day 21**	18.68	22.24	21.98	26.24		24.00	22.94
**Day 24**						24.32	22.32
**Day 25**				26.38	26.00		
**Day 26**				25.94	25.20	23.76	22.32
**Day 27**				26.72	25.68		
**Day 28**				26.34	25.36	23.76	22.08
**Day 29**	18.76	21.44		26.10	25.06		
**Day 30**				25.58	25.20	23.80	22.16
**Day 31**	19.66		22.40	25.38	25.76		
**Day 32**				25.52	25.06	24.48	22.16
**Day 33**		21.44	22.28	26.10	25.04		
**Day 34**	19.66	21.12		25.48	24.96	24.74	22.48

B. Low iron diet.

	Round 2	Round 3	Round 4	Round 5	Round 6a	Round 6b
**Day 0**	21.06	21.64		25.65	23.82	22.28
**Day 8**			26.70			
**Day 10**						
**Day 16**			26.83			
**Day 21**	23.30	22.84	27.13		25.20	21.96
**Day 24**					25.78	21.74
**Day 25**			27.57	25.80		
**Day 26**			27.05	26.78	25.44	21.84
**Day 27**			27.50	26.73		
**Day 28**			26.98	26.90	25.42	21.74
**Day 29**	22.96		27.08	26.55		
**Day 30**			27.25	26.70	25.94	21.98
**Day 31**		23.18	26.93	25.83		
**Day 32**			26.55	25.35	25.28	22.04
**Day 33**	23.28		26.55	25.50		
**Day 34**	24.06	23.60	26.45	24.83	25.44	22.62

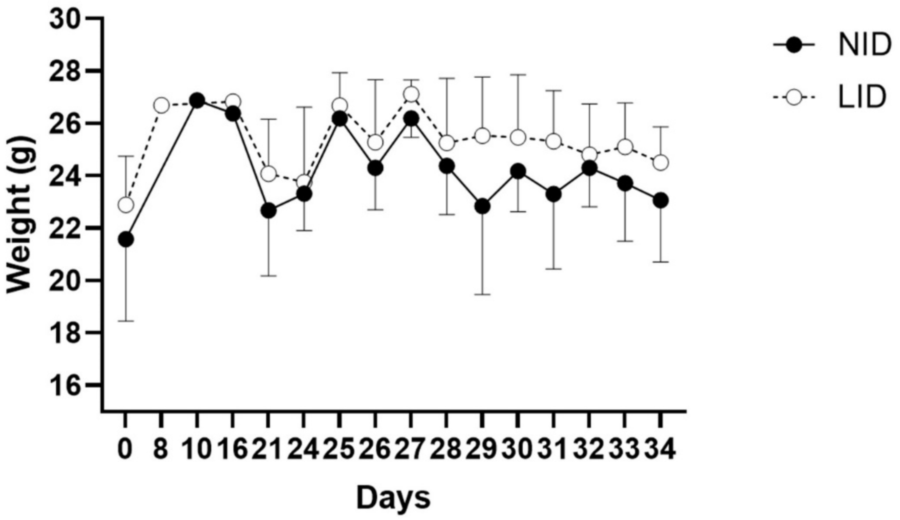

NID: normal iron diet, LID: low iron diet

Supplementary Table S3: Quantification of iron content (A), oxidative stress (B) and lipid peroxidation (C) in selected paws at day 34 from mice fed a normal iron diet or a low iron diet. The results are presented graphically below the tables. The term "round" refers to a single independent experiment. In round 6, two cages were operated in parallel for each condition, which is differentiated by "a" and "b".

A.

Normal iron diet			Low iron diet			
CAS	*Fe^{2+}*	*Fe^{3+}*	*CAS*	*Fe^{2+}*	*Fe^{3+}*	
1	30.85	14.09	1	66.62		Round 6a
0	51.06	7.05	1	23.27	6.12	
0	44.81	10.50	0	65.82	5.32	
1	64.62	1.86	1	44.14	17.82	
1	26.59	4.52	0	39.76	22.07	
0	28.19	4.92	0	25.40	10.24	Round 6b
0	44.28	5.05	1	34.17	8.51	
1	43.61		1	49.20	18.22	
1	69.41	7.85	1	22.74	6.38	
3	27.66	10.90	1	32.18	3.86	

$Fe^{2+}$and Fe^{3+} are depicted as µg per g tissue. CAS: clinical arthritis score

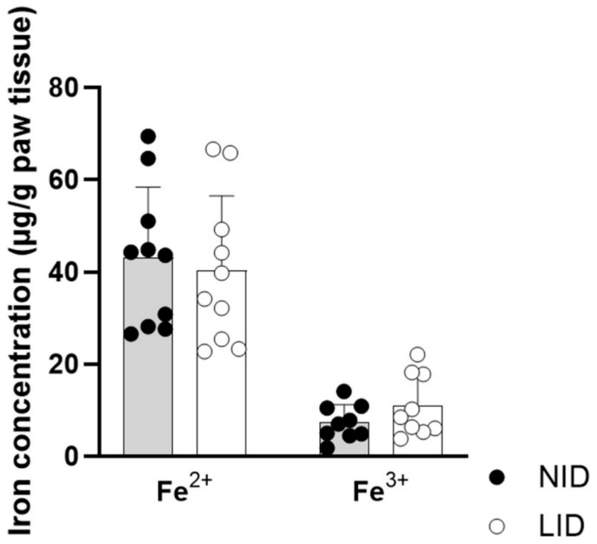

NID: normal iron diet, LID: low iron diet

B.

Normal iron diet				Low iron diet				
CAS	*GSH*	*GSSG*	*GSH/GSSG (~ratio)*	*CAS*	*GSH*	*GSSG*	*GSH/GSSG (~ratio)*	
1	0.55	0.98	0.56 (1:1.5)	1	0.41	1.43	0.29 (1:3)	Round 6a
0	0.63	1.29	0.49 (1:2)	1	0.54	1.22	0.44 (1:2.5)	
0	0.66	1.11	0.60 (1:1.5)	0	0.41	1.11	0.37 (1:2.5)	
1	1.01	1.05	0.96 (1:1)	1	0.74	0.99	0.75 (1:1.3)	
1	1.03	0.98	1.05 (1:1)	0	0.47	1.40	0.34 (1:3)	
0	0.73	1.28	0.57 (1:1.5)	0	0.44	1.28	0.34 (1:3)	Round 6b
0	0.14	0.71	0.20 (1:5)	1	0.57	1.22	0.47 (1:2)	
1	1.09	1.12	0.97 (1:1)	1	0.91	1.28	0.71 (1:1.4)	
1	0.74	1.12	0.66 (1:1.5)	1	0.22	1.51	0.15 (1:5)	
3	0.62	1.31	0.47 (1:2)	1	1.22	1.17	1.04 (1:1)	

GSH and GSSG are depicted as µM per g tissue. CAS: clinical arthritis score, GSH: reduced glutathione, GSSG: oxidized glutathione

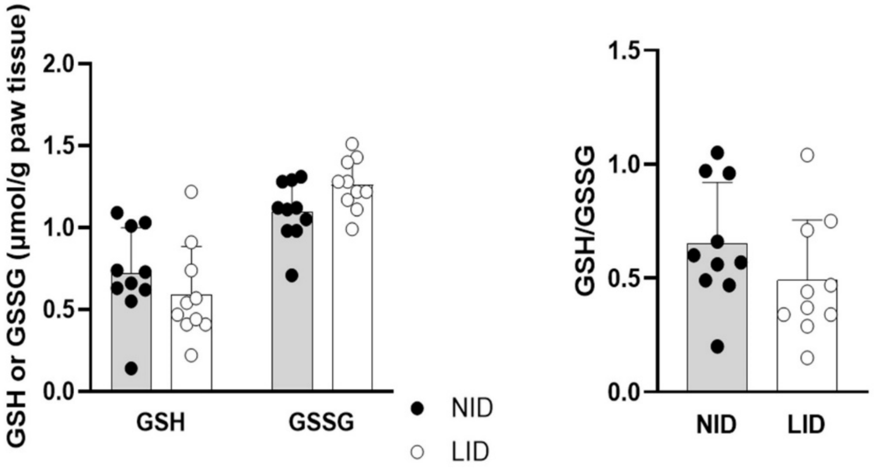

GSH: reduced glutathione, GSSG: oxidized glutathione, NID: normal iron diet, LID: low iron diet

C.

Normal iron diet		Low iron diet		
CAS	*MDA*	*CAS*	*MDA*	
1	50.83	1	53.55	Round 6a
0	36.50	1	21.55	
0	137.16	0	30.16	
1	44.38	1	21.38	
1	66.16	0	25.22	
0	38.88	0	30.61	Round 6b
0	46.66	1	42.55	
1	44.77	1	77.00	
1	32.55	1	23.61	
3	13.44	1	42.88	

MDA is depicted as nmol per mg. CAS: clinical arthritis score, MDA: malondialdehyde. For NID vs. LID p=0.25.

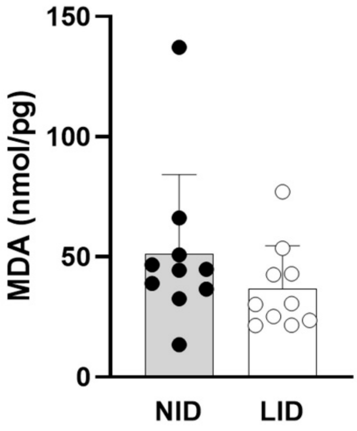

MDA: malondialdehyde, NID: normal iron diet, LID: low iron diet

Supplementary Table S4: Relative gene expression of master regulator genes involved in (A) iron metabolism and (B) ferroptosis in selected paws at day 34 from mice under normal iron diet or low iron diet using the 2(-Delta C(T)) method. The results are presented graphically below the tables. The term “round” refers to a single independent experiment. In round 6, two cages were operated in parallel for each condition, which is differentiated by “a” and “b”.

A. Relative gene expression of FTH1, TFRC, FPN1, VEGF and SDF1

	**Normal iron diet**		**Low iron diet**	
Gene	2(-Delta C(T))	CAS	2(-Delta C(T))	CAS
FTH1	**Round 4**		**Round 4**	
	0.052515	2	0.99274	1
	10.13349	2	2.022516	1
	3.646186	2	0.001084	1
	11.6005	2	11.7771	1
	0.135457	3		
	**Round 5**		**Round 5**	
	0.60039	1	4.280677	3
	0.906041	3	15.19163	1
	18.43435	1	0.298324	1
	0.036672	1	2.01016	1
	0.891844	2		
	**Round 6a**		**Round 6a**	
	113.4822	1	6.323192	3
	1.307808	2	5.445164	0
	0.075545	2	7.761434	0
	0.034889	0	0.098198	0
	0.182534	0	5.224502	0
	**Round 6b**		**Round 6b**	
	6.429885	2	0.006971	0
	0.366909	2	6.61629	0
	4.357648	3	1.122475	0
	1.383708	1	0.179834	0
	0.984545	2	0.783455	0
TFRC	**Round 4**		**Round 4**	
	9.515913	2	0.360692	1
	1.876179	2	1.023598	1
	7.759326	2	1.608761	1
	0.166692	3		
	**Round 5**		**Round 5**	
	0.47178	1	1.65812	3
	0.112945	3	4.324658	1
	4.988691	1	0.161552	1
	0.162908	2	1.453321	1
	**Round 6a**		**Round 6a**	
	2.160937	1	1.776302	3
	1.699111	2	0.582064	0
	0.161209	2	2.270829	0
	0.864524	0	0.171863	0
			1.968405	0
	**Round 6b**		**Round 6b**	
	5.757753	2	2.236388	0
	1.000517	2	1.839951	0
	1.51366	3	0.20819	0
	0.53528	1	1.469664	0
	0.41868	2		
FPN1	**Round 4**		**Round 4**	
	0.184884	2	0.440662	1

	3.057489	2	0.813413	1
	0.983298	2	0.245469	1
	6.118763	2	3.4771	1
	0.342052	3		
	**Round 5**		**Round 5**	
	1.279375	1	0.996979	3
	0.516985	3	5.695489	1
	8.141583	1	0.308699	1
	0.220468	1	1.864732	1
	0.724043	2		
	**Round 6a**		**Round 6a**	
	1.137336	1	2.934641	3
	1.224414	2	1.10364	0
	0.148329	2	2.852536	0
	1.019717	0	0.258135	0
	1.020624	0	1.418387	0
	**Round 6b**		**Round 6b**	
	4.145498	2	0.143329	0
	0.831311	2	6.052913	0
	3.991161	3	1.661272	0
	0.844824	1	0.174537	0
	0.40032	2	1.175216	0
VEGF	**Round 4**		**Round 4**	
	0.367676	2	0.796655	1
	1.864795	2	1.047319	1
	0.921281	2	0.064694	1
	4.061129	2	4.181434	1
	1.24903	3		
	**Round 5**		**Round 5**	
	0.498866	1	3.245595	3
	0.776395	3	2.56349	1
	2.892731	1	0.329721	1
	0.377394	1	1.615075	1
	0.73811	2		
	**Round 6a**		**Round 6a**	
	13.33958	1	1.684028	3
	1.701998	2	1.010347	0
	0.430776	2	1.570384	0
	0.482852	0	0.497295	0
	0.338412	0	1.733329	0
	**Round 6b**		**Round 6b**	
	1.544382	2	0.328002	0
	0.734112	2	2.229211	0
	1.114345	3	1.413787	0
	0.458773	1	0.362328	0
	1.079574	2	1.159217	0
SDF1	**Round 4**		**Round 4**	
	0.096265	2	0.682499	1
	4.961948	2	1.375416	1
	3.329794	2	0.037784	1
	4.171905	2	3.816341	1
	0.357934	3		
	**Round 5**		**Round 5**	
	0.87391	1	1.671161	3
	0.82371	3	4.164265	1
	3.074519	1	0.630369	1
	0.070702	1	1.684071	1
	2.690764	2		
	**Round 6a**		**Round 6a**	
	14.24845	1	8.799753	3
	0.874616	2	2.172325	0

	0.151171	2	3.150226	0
	0.340492	0	0.130634	0
	0.362174	0	1.661776	0
	**Round 6b**		**Round 6b**	
	10.2779	2	0.117085	0
	0.346007	2	5.749161	0
	8.017927	3	0.789001	0
	0.569126	1	0.356756	0
	0.265254	2	0.40372	0

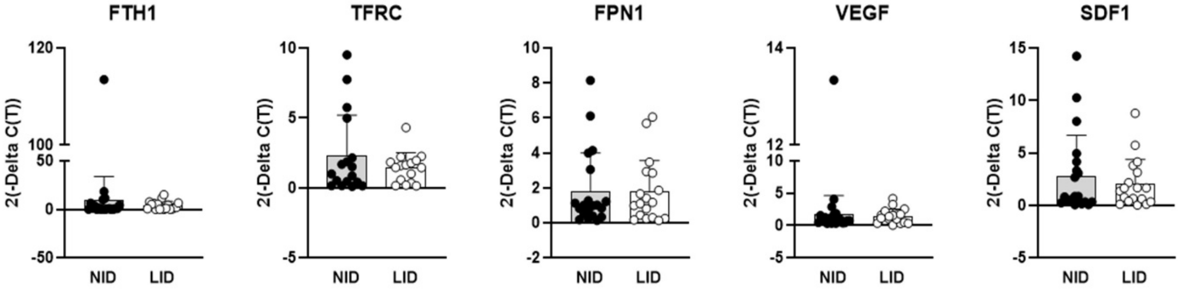

CAS: clinical arthritis score, FTH1: ferritin heavy chain 1, TFRC: transferrin receptor protein 1, FPN1: ferroportin-1, VEGF: vascular endothelial growth factor, SDF1: stromal cell-derived factor 1, NID: normal iron diet, LID: low iron diet

B. Relative gene expression of GPX4, ACSL4, SLC7A11 and SLC3A2

	**Normal iron diet**		**Low iron diet**	
Gene	2(-Delta C(T))	CAS	2(-Delta C(T))	CAS
GPX4	**Round 4**		**Round 4**	
	0.220572	2	1.184577	1
	5.515286	2	1.585844	1
	3.581581	2	0.028559	1
	6.089891	2	9.536992	1
	0.271714	3		
	**Round 5**		**Round 5**	
	1.215385	1	1.85961	3
	0.163006	3	13.04019	1
	8.283931	1	0.039897	1
	0.228675	1	2.020077	1
	0.369582	2		
	**Round 6a**		**Round 6a**	
	16.28695	1	1.537431	3
	2.241009	2	1.10543	0
	0.154659	2	3.973623	0
	0.25422	0	0.308296	0
	0.320202	0	2.192501	0
	**Round 6b**		**Round 6b**	
	1.922937	2	0.077761	0
	1.197464	2	4.331248	0
	1.913621	3	1.769526	0
	0.754269	1	0.240775	0
	0.654785	2	1.526637	0
ACSL4	**Round 4**		**Round 4**	
	11.13821	2	0.486835	1
	1.977995	2	1.848839	1
	3.598122	2	0.011157	1
	0.020038	3	4.619897	1
	**Round 5**		**Round 5**	
	0.617896	1	2.740061	3
	0.324042	3	8.848993	1
	9.165251	1	0.400129	1
	0.343061	2	2.221762	1
	**Round 6a**		**Round 6a**	
	17.67783	1	2.850439	3
	1.977793	2	0.774189	0
	0.121194	2	2.675425	0
	0.162572	0	0.05121	0
	0.037448	0	2.618581	0
	**Round 6b**		**Round 6b**	
	2.907228	2	3.600315	0
	1.33655	2	1.200128	0
	2.442841	3	0.252003	0
	9.291851	1	1.159994	0
	0.439521	2		
SLC7A11	**Round 4**		**Round 4**	
	1.198988	2	0.35655	1
	1.59073	2	2.232254	1
	0.863825	2	0.029347	1
	3.87783	2	4.223794	1
	4.050223	3		
	**Round 5**		**Round 5**	
	0.076879	1	1.709946	3
	0.92781	3	1.510964	1
	0.842451	1	0.368804	1
	0.755426	1	10.63743	1

	0.85132	2		
	**Round 6a**		**Round 6a**	
	94.3733	1	0.689447	3
	1.839551	2	0.514069	0
	0.338992	2	1.765991	0
	0.391821	0	0.434866	0
	0.118234	0	1.642179	0
	**Round 6b**		**Round 6b**	
	2.023809	2	0.656378	0
	0.353555	2	2.34996	0
	1.8436	3	1.757737	0
	0.228728	1	0.598017	0
	1.215641	2	1.379849	0
SLC3A2	**Round 4**		**Round 4**	
	0.020132	2	0.020104	1
	9.37878	2	0.441502	1
	2.888462	2	1.501537	1
	14.85697	2	7.392065	1
	0.257944	3		
	**Round 5**		**Round 5**	
	1.247842	1	0.110121	3
	0.769818	3	7.923386	1
	21.92408	1	24.75596	1
	0.021245	1	0.469907	1
	1.06934	2		
	**Round 6a**		**Round 6a**	
	26.37891	1	3.09952	3
	1.852454	2	1.120301	0
	0.14495	2	4.565421	0
	0.202767	0	0.135569	0
	0.279325	0	3.657822	0
	**Round 6b**		**Round 6b**	
	3.69618	2	0.048685	0
	0.773233	2	8.098519	0
	3.604028	3	1.310162	0
	0.669324	1	0.22408	0
	0.361561	2	1.098948	0

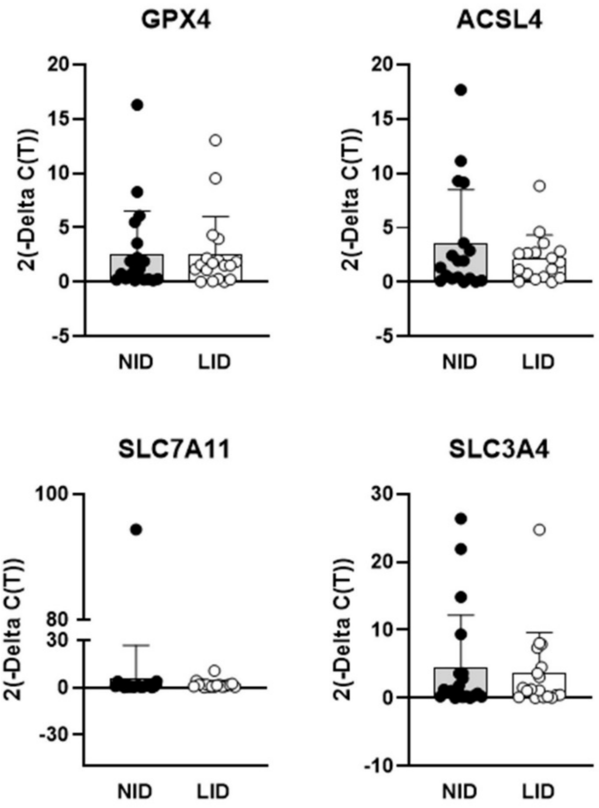

CAS: clinical arthritis score, GPX4: glutathione peroxidase 4, ACSL4: acyl-CoA synthetase long chain family member 4, SLC7A11: solute carrier family 7 member 11, SLC3A2: solute carrier family 3 member 2, NID: normal iron diet, LID: low iron diet
